# Protist predation selects for the soil resistome

**DOI:** 10.1093/ismejo/wrad007

**Published:** 2024-01-10

**Authors:** Gaofei Jiang, Chen Liu, Wu Xiong, Qirong Shen, Zhong Wei

**Affiliations:** Key Lab of Organic-based Fertilizers of China, Jiangsu Provincial Key Lab for Solid Organic Waste Utilization, Nanjing Agricultural University, 1 Weigang, Xuanwu District, Nanjing 210095, China; Key Lab of Organic-based Fertilizers of China, Jiangsu Provincial Key Lab for Solid Organic Waste Utilization, Nanjing Agricultural University, 1 Weigang, Xuanwu District, Nanjing 210095, China; Key Lab of Organic-based Fertilizers of China, Jiangsu Provincial Key Lab for Solid Organic Waste Utilization, Nanjing Agricultural University, 1 Weigang, Xuanwu District, Nanjing 210095, China; Key Lab of Organic-based Fertilizers of China, Jiangsu Provincial Key Lab for Solid Organic Waste Utilization, Nanjing Agricultural University, 1 Weigang, Xuanwu District, Nanjing 210095, China; Key Lab of Organic-based Fertilizers of China, Jiangsu Provincial Key Lab for Solid Organic Waste Utilization, Nanjing Agricultural University, 1 Weigang, Xuanwu District, Nanjing 210095, China

**Keywords:** protist predation, selection pressure, antibiotic resistance, soil bacterial community, One Health

## Abstract

A key aspect of “One Health” is to comprehend how antibiotic resistomes evolve naturally. In this issue, Nguyen and colleagues pioneered an *in situ* investigation on the impact of protist predations on the soil microbial community and its antibiotic resistance genes (ARGs). They found that bacterivorous protists consistently increased the abundance of ARGs, such as tetracycline resistant genes. Indeed, antibiotic production is a common strategy for bacteria to evade protist predation. The rise of ARGs can be explained by the balance between antibiotic producers and resisters shaped by predatory selection. This work suggests that ARG enrichment due to biotic interactions may be less worrisome than previously thought. Unless, these ARGs are carried by or disseminated among pathogens. Therefore, it is essential to monitor the occurrence, dissemination and pathogenic hosts of ARGs, enhancing our capacity to combat antibiotic resistance.

## Main

Antibiotic resistance poses a growing global threat to public health and is a pivotal concern in the context of a “One Health” framework [1], which emphasizes the interconnectedness of human health with the well-being of other ecosystem components. A particular concern is the potential emergence of antibiotic resistance genes (ARGs) that may sweep across microbiomes [2]. ARGs can spread from soil environments into the food chain via plants, thereby posing substantial risks to both livestock and human health [3]. Although the ARG accumulation in soils is often attributed to the effect of human activities, such as antibiotics pollution. Yet, the ARG dynamics may be also driven by interactions between bacteria and their natural enemies such as bacteriophages, fungi, competing bacteria and protists. Bacterivorous protists deserve a special attention and have been speculated to enrich ARGs in soils [4], they are one of the primary consumer of soil bacteria [5]. To survive in a complex soil environment, bacteria evolve and produce more antibiotics [6] to avoid predation. Consequently, this selective pressure by protists may lead to the dominance of antibiotic-producing bacteria (APB) within soil bacterial communities. This in turn may create a selective pressure for antibiotics resistance, increasing the overall soil ARG abundance. Some studies have tested interactions between model bacterial strains and protists under *in vitro* conditions. However, there remains a lack of compelling evidence regarding the impact of soil protists on antibiotic resistance in soils at the community level *in situ.*

In a new study [7], Nguyen and colleagues explored the role of protist communities in shaping the abundance and diversity of the resistome in soils. They achieved this by profiling the resistome dynamics in soil microcosms subject to low, medium, and high predatory pressure from protists. This study revealed that soil protists significantly enhanced the development of soil ARGs at the community level. In particular, the enriched ARGs were highly associated with multidrug and tetracycline efflux pumps, as well as antibiotic deactivation. Beyond alterations in bacterial community composition, protists also raised the relative abundance of some well-reported APB, such as *Mycobacterium*, *Nocardia*, and *Streptomycetaceae*. In summary, the study makes a substantial contribution to our understanding of how protistan predation exerts top-down manipulations on bacterial communities, consequently fostering the proliferation of APB and antibiotic-resistant bacteria (ARB). These two populations generally maintain a dynamic equilibrium in the soil microbial community. Antibiotics production is costly and only pays off under selective pressures [8]. Predatory protists, as well as other soil predators (e.g. predatory nematodes or even bacteria), impose significant selective pressures on microbial communities and favour APB, and as a knock-on effect ARGs. Additionally, niche competition (e.g. for nutrients and space) likewise drives antibiotic production and hence would enrich the levels of ARGs. Biotic interactions can promote the evolution of resistance through diverse mechanisms other than selecting ARB via antagonism. For instance, fungi can promote the spread of ARGs by increasing community spatial intermixing [9].

Antibiotic resistance is a common survival strategy of microbes under natural biotic interactions in soils. In this context, the enrichment of ARGs derived from naturally biotic interactions *per se* may be less worrisome to One Health than previously thought. It is also important to acknowledge that it is neither feasible nor practical to eliminate ARGs in dynamic and complex ecosystems such as soil and water. Instead, to curb antibiotic resistance, we should revisit the proliferation of ARGs and establish surveillance systems to survey and monitor the distribution of ARGs in pathogens infecting humans, animals, and plants in the environment [10]. The surveillance framework should prioritize the risk of ARGs based on their possibility to disseminate across ecosystems, their potential acquisition by pathogens, and the degree of resistance they confer to counteract antibiotic exposures [11]. Thus, it becomes imperative to establish consensus approaches to identify novel ARGs against antibiotics and track the transmission and pathogenic hosts of ARGs in environments.

It is crucial to evaluate the relative contribution of natural biotic interactions and anthropogenic disturbances to the enrichment of risk ARGs. For instance, microbial adaptation to antimicrobials such as volatile compounds is accompanied by cross-tolerance to antibiotics [12]. However, little is known about whether antagonistic volatiles are related to ARG accumulation in multitrophic interactions. To fill these knowledge gaps, it is essential to advance and develop novel powerful approaches, including metagenomics, isotope labelling techniques, single-cell technologies, and bioinformatic tools. Indeed, recent advancements in experimental techniques and computational tools have opened up novel avenues for ARG surveillance. For instance, the integration of microfluidics and single-cell sequencing allows us to investigate the horizontal gene transfer of ARGs among bacterial cells within a complex microbiome [13]. This, along with growing knowledge on genomic signatures of ARGs [14] and pathogen identification by deep learning [15], offers opportunities to reconstruct the exchange network of ARGs in soils. Tracking the environmental spread of high-risk ARGs, especially from non-pathogenic to pathogenic bacteria, empowers the development of targeted strategies to combat antibiotic resistance.
